# Development and Characterization of Highly Stable Silver NanoParticles as Novel Potential Antimicrobial Agents for Wound Healing Hydrogels

**DOI:** 10.3390/ijms23042161

**Published:** 2022-02-15

**Authors:** Alessio Massironi, Albina Ribeiro Franco, Pedro Sousa Babo, Dario Puppi, Federica Chiellini, Rui L. Reis, Manuela Estima Gomes

**Affiliations:** 1 Department of Chemistry and Industrial Chemistry, University of Pisa, UdR INSTM-Pisa, Via G. Moruzzi 13, 56124 Pisa, Italy; alessio.massironi@unimi.it (A.M.); dario.puppi@unipi.it (D.P.); federica.chiellini@unipi.it (F.C.); 2 3B’s Research Group, I3Bs—Research Institute on Biomaterials, Biodegradables and Biomimetics, University of Minho, Headquarters of the European Institute of Excellence on Tissue Engineering and Regenerative Medicine, AvePark—Parque de Ciência e Tecnologia, Zona Industrial da Gandra, 4805-017 Guimaraes, Portugal; albina.franco@i3bs.uminho.pt (A.R.F.); pedro.babo@i3bs.uminho.pt (P.S.B.); rgreis@i3bs.uminho.pt (R.L.R.); 3 ICVS/3B’s—PT Government Associate Laboratory, 4805-017 Guimaraes, Portugal

**Keywords:** nanotechnology, silver nanoparticles, hydrogels, polysaccharides, ulvan, cellulose nanocrystals, wound healing

## Abstract

Recurrent microbial infections are a major cause of surgical failure and morbidity. Wound healing strategies based on hydrogels have been proposed to provide at once a barrier against pathogen microbial colonization, as well as a favorable environment for tissue repair. Nevertheless, most biocompatible hydrogel materials are more bacteriostatic than antimicrobial materials, and lack specific action against pathogens. Silver-loaded polymeric nanocomposites have efficient and selective activity against pathogenic organisms exploitable for wound healing. However, the loading of metallic nanostructures into hydrogels represents a major challenge due to the low stability of metal colloids in aqueous environments. In this context, the aim of the present study was the development of highly stable silver nanoparticles (AgNPs) as novel potential antimicrobial agents for hyaluronic acids hydrogels. Two candidate stabilizing agents obtained from natural and renewable sources, namely cellulose nanocrystals and ulvan polysaccharide, were exploited to ensure high stability of the silver colloid. Both stabilizing agents possess inherent bioactivity and biocompatibility, as well as the ability to stabilize metal nanostructures thanks to their supramolecular structures. Silver nitrate reduction through sodium borohydride in presence of the selected stabilizing agents was adopted as a model strategy to achieve AgNPs with narrow size distribution. Optimized AgNPs stabilized with the two investigated polysaccharides demonstrated high stability in phosphate buffer saline solution and strong antimicrobial activity. Loading of the developed AgNPs into photocrosslinked methacrylated hyaluronic acid hydrogels was also investigated for the first time as an effective strategy to develop novel antimicrobial wound dressing materials.

## 1. Introduction

Post-surgical wound infections are a major burden worldwide. Hydrogels have been investigated as suitable materials to prevent post-surgical bacterial infections, in the form of wound dressing patches or coatings of medical devices, acting as a barrier against pathogen microbial colonization, and providing a favorable environment for wound healing [1]. Indeed, hydrogels with antimicrobial properties are currently included in different medical tools such as bandages, hard implants for bone and teeth reconstruction, personal care products, sanitizers, and disinfectants to be applied to open wounds [2]. Hydrogel-based devices can provide to the wounds a moist environment while absorbing extensive exudate effectively [3]. Moreover, polymeric hydrogels can present a macromolecular structure similar to native extracellular matrix (ECM) promoting the healing processes, and can be easily loaded with antimicrobial agents and growth factors [3]. In particular, the antimicrobial activity can be provided through the loading of biocidal agents as well as by the inherent biocidal properties of the polymer matrix [4]. Antimicrobial hydrogels are commonly divided into four main categories: hydrogels with inherent antimicrobial properties [5], antibiotic-loaded hydrogels [6], biological extract-loaded hydrogels [7,8] and metal nanoparticles-loaded hydrogels [9,10], the latter displaying the strongest bioactivity [11]. Moreover, metal nanoparticles loading demonstrated to positively influence the mechanical properties of the obtained hydrogel as well as its optical features [12,13], which solves one of the major causes of hydrogel-wound dressing failure.

Metal nanostructures can be loaded within the hydrogel as crosslinking agents of the polymeric matrix, or be entrapped into it through weak interactions [14,15,16,17]. Among metals, silver is the most investigated one to this purpose thanks to its strong antibacterial property, relatively low toxicity and moderate synthesis costs [18,19,20]. However, the low colloidal stability of metal nanostructures could lead to the formation of an inactive and even toxic device, whose application into the human body may cause severe side effects to the patients [17]. For this reason, silver nanoparticles (AgNPs) stabilization still represents a major challenge for the scientific community [21]. Indeed, only few studies have reported an efficient loading of stable metal nanostructures within hydrogels, typically employing chitosan as polymeric matrix [22,23].

The aim of the present study was the development of highly stable AgNPs obtained through chemical reduction in the presence of either cellulose nanocrystals (CNCs) or ulvan polysaccharide as novel antimicrobial agents for hydrogels preparations. Methacrylated-hyaluronic acid (Met-HA) was selected as photoactivable based-material since it represents one of the most promising biocompatible polymers for hydrogel preparation [24,25,26,27]. Indeed, HA and its derivates have been demonstrated to positively affect cellular response in terms of cell attachment and growth [28]. Several studies demonstrated HA- hydrogels to be optimal tools for wound healing treatment by inducing mesenchymal and epithelial cell migration and differentiation, improved angiogenesis, and collagen deposition [29]. Methacrylated-HA presents enhanced robustness in comparison to unmodified HA-hydrogels, and different degradation rates can be achieved by tuning the crosslinking degree, for instance by varying the number of methacrylate groups along the HA chain [30].

In this study, particular attention was given to the selection of a stabilizing agent preventing AgNPs’ aggregation and loss of colloidal conformation, while enhancing nanosystem biocompatibility. In this context, both selected polysaccharides, namely ulvan and cellulose nanocrystals (CNC), have been demonstrated to be biocompatible and to have relevant tissue healing properties. Moreover, their use as stabilizing agents of metallic nanoparticles has been recently confirmed, supporting the possible suitability of these macromolecules to be employed as biomaterials [31,32,33,34].

In recent years, ulvan polysaccharide has received particular attention by virtue of its unique physico-chemical features and widespread availability [35]. The chemical composition of ulvan is largely variable and is usually dependent on different factors, such as the harvesting season, growth conditions, taxonomic origins and the post-collection treatment of the biomass. Its chemical structure is formally represented as the sequence of two disaccharide repeating units, i.e., β-D-glucuronic acid (1→4)-α-L-rhamnose 3-sulphate (A3s) and α-L-iduronic acid (1→4)-α-L-rhamnose 3-sulphate (B3s) [19,36]. Indeed, the strong bioactivity and antioxidant activity of ulvan seem to be correlated with the distribution and degree of substitution of sulfate groups along the polymeric backbone [37,38]. Moreover, ulvan displays an interesting chemical versatility due to its amphiphilic character, provided by both hydrophilic and hydrophobic groups within its repeating units [36,39,40]. CNCs are receiving considerable interest due to their unique features, such as the renewable nature of cellulose, and a combination of biological and physical properties provided by its crystalline structure, nanometric size and high surface area [41]. Indeed, CNCs have recently been shown to significantly enhance the stability and mechanical performance of hydrogels and endow them with interesting biological features [25]. CNC hydrophilicity is provided by the presence of several hydroxyl groups on its surface allowing easy chemical functionalization and providing most of its biological features [42].

In this research activity, both polysaccharides were selected to design stable AgNPs tailored to the formulation of antimicrobial hydrogels with enhanced structural stability and bioactivity. The developed metallic colloids were characterized by UV spectroscopy, Dynamic Light Scattering (DLS), Zeta potential, X-ray diffraction analysis (XRD) analysis, Scanning Transmission Electron Microscopy (STEM), Atomic Force Microscopy (AFM) and Fourier-transform infrared spectroscopy (FT-IR). A preliminary investigation over AgNPs loading within methacrylated HA hydrogels was conducted in order to preliminarily assess the effect of the AgNPs in hydrogels gelification and stability.

## 2. Results

### 2.1. AgNPs Preparation and Characterization

**Figure 1:**
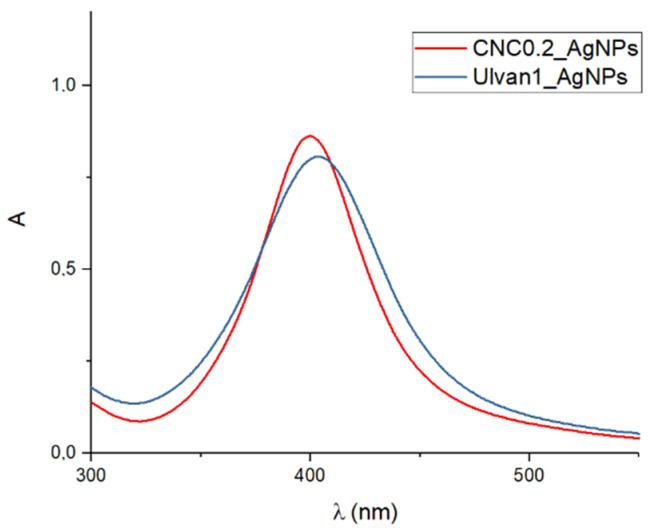
UV-vis spectra comparison between selected AgNPs stabilized by CNC and ulvan polysaccharide.

AgNPs were synthesized following a conventional method employing NaBH_4_ as reducing agent, in presence of a stabilizer: ulvan or CNCs. The selected chemical method was optimized in order to adopt the same synthesis approach for all formulations thus reducing the influence of the adopted experimental conditions. The comparison of the surface plasmon resonance band (SPR) of all developed formulations recorded at different time points, allowed us to identify the most stable colloids, as well as to identify comparable colloidal systems in terms of inorganic core shape and size. Indeed, as reported in Figure 1, CNC0.2_AgNPs ([CNC] = 0.2 mg/mL) and Ulvan1_AgNPs ([ulvan] = 1 mg/mL) displayed an almost superimposable SPR band suggesting a similar inorganic core size and shape. 

The DLS size distribution and average diameters of the most promising formulations in terms of colloidal stability are reported in Table 1. The colloidal stability of selected silver nanoparticles (AgNPs) formulations was also predicted through zeta potential analysis (pH 7.4) (Table 1), evidencing a high negative value for cellulose nanocrystals silver nanoparticles [CNC: 0.2 mg/mL] (CNC0.2_AgNPs) and ulvan silver nanoparticles [ulvan: 1 mg/mL] (Ulvan1_AgNPs) provided by the polysaccharide shell. Zeta potential analysis predicted a good stability of both AgNPs as revealed by the assessment of a large negative value likely provided by the presence of a polysaccharide. Indeed, it is generally accepted that for zeta potential values above 30 mV or below −30 mV, colloidal systems tend to be stable because of the electrostatic repulsions occurring between the nanoparticles that prevent them from collapsing to the aggregated form [43].

Accordingly, among obtained colloidal suspensions CNC0.2_AgNPs [CNC: 0.2 mg/mL] and Ulvan1_AgNPs [ulvan: 1 mg/mL] were selected as model AgNPs since they demonstrated higher colloidal stability and similar inorganic core structure.

The DLS analysis revealed the presence of multimodal size distribution for CNC0.2_AgNPs (mean diameter 188.9 nm) and monomodal size distribution for Ulvan1_AgNPs (mean diameter 70.8 nm) as reported by DLS size distribution graph, in which different particles population have been observed. The high polydispersion of the average particles’ size displayed by CNC0.2_AgNPs is probably due to the rod-like shape of CNCs which did not allow measuring the correct diameter of the nanorods by means of DLS analysis (Table 1).

**Figure 2:**
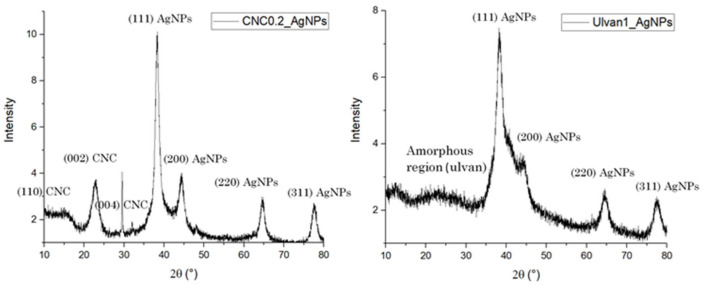
XRD pattern of CNC0.2_AgNPs and Ulvan1_AgNPs.

The dimension of the inorganic core of selected AgNP formulations was estimated by XRD analysis. The XRD pattern of both synthesized AgNPs showed the presence of all diagnostic crystalline planes of silver nanospheres: (111) at 36°, (200) at 46°, (220) at 64° and (311) at 78° (Figure 2). The crystalline conformation of cellulose nanocrystal seems not to be affected by the interaction with the AgNPs showing a strong peak at 15°, 22° and 32° corresponding to crystal plane (110), (002) and (004), respectively. The average diameter of the inorganic core of CNC0,2_AgNPs and Ulvan1_AgNPs calculated by using Debye–Scherrer’s equation, was found to be 8.9 and 7.8 nm, respectively. I_(002) is the maximum intensity of peak diffraction at 2θ = 22° corresponding to the crystallinity region of (002) plane, while I_amorphous corresponds to the amorphous region at 2θ = 16–19°. The estimated cellulose crystallinity index calculated for CNC0.2_AgNPs is 60.2%, confirming the high percentage of cellulose in crystalline conformation even after the reduction process and its stabilizing activity during AgNPs formation.

**Figure 3:**
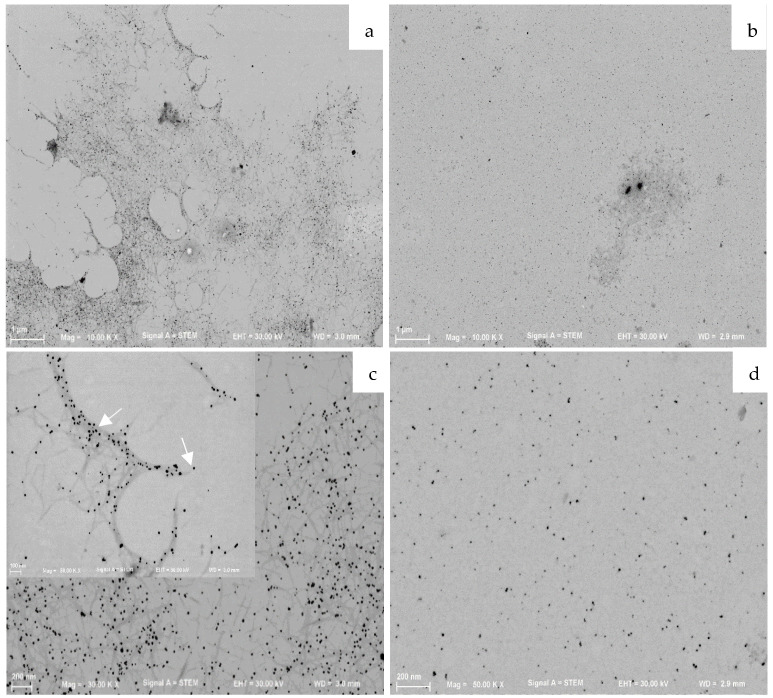
STEM images of CNC0.2_AgNPs (**a**,**c**) and Ulvan1_AgNPs (**b**,**d**). (**a**) CNC0.2_AgNPs; (**b**) Ulvan1_AgNPs; (**c**) CNC0.2_AgNPs; (**d**) Ulvan1_AgNPs.

The average size of the inorganic core of CNC0.2_AgNPs and Ulvan1_AgNPs calculated through STEM analysis resulted was 11.7 ± 2.9 and 9.5 ± 1.8 nm, respectively (Figure 3).

**Figure 4:**
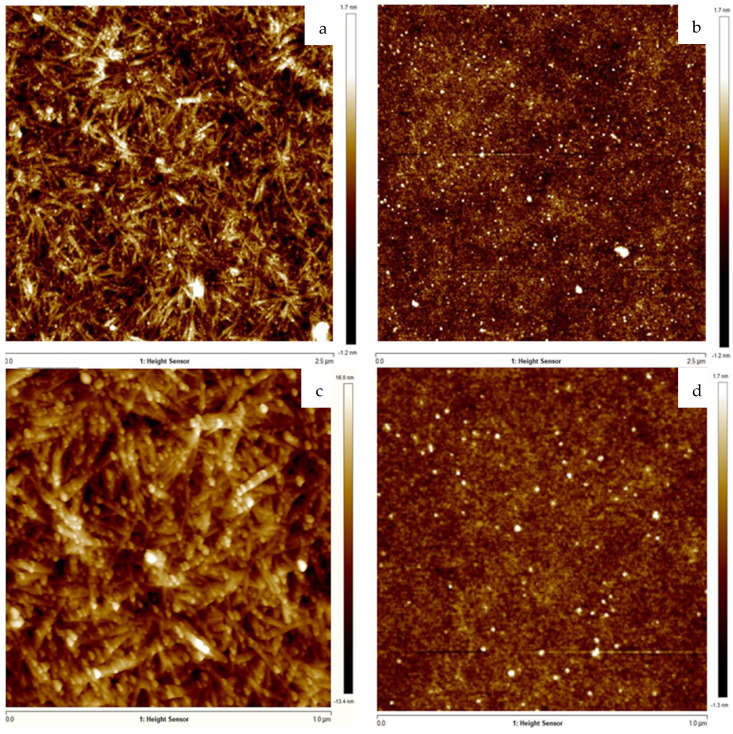
AFM images of CNC0.2_AgNPs (**a**,**c**) and Ulvan1_AgNPs (**b**,**d**). (**a**) CNC0.2_AgNPs; (**b**) Ulvan1_AgNPs; (**c**) CNC0.2_AgNPs; (**d**) Ulvan1_AgNPs.

Moreover, STEM analysis allowed determining the average size and shape of CNCs with a length range of 214.7 ± 34.0 nm (Figure 3a,c) and their interaction with AgNPs. No silver particles were observed in absence of CNCs, while all AgNPs seem to be adsorbed on the CNCs surface. On the contrary, STEM and AFM analysis (Figure 3b,d; Figure 4b,d) did not allow observing the presence of ulvan coating, due to the amorphous conformation of the polysaccharide. However, a hypothesized core-shell conformation of ulvan-AgNPs has been supported by DLS analysis, where only one homogeneous particle population of 70.8 nm was observed while the inorganic silver core resulted of less than 10 nm.

Finally, the hypothesized interaction between CNCs and AgNPs was confirmed through the AFM analysis. The metal colloid was found to be adsorbed on the CNCs surface (Figure 4a,c).

**Figure 5:**
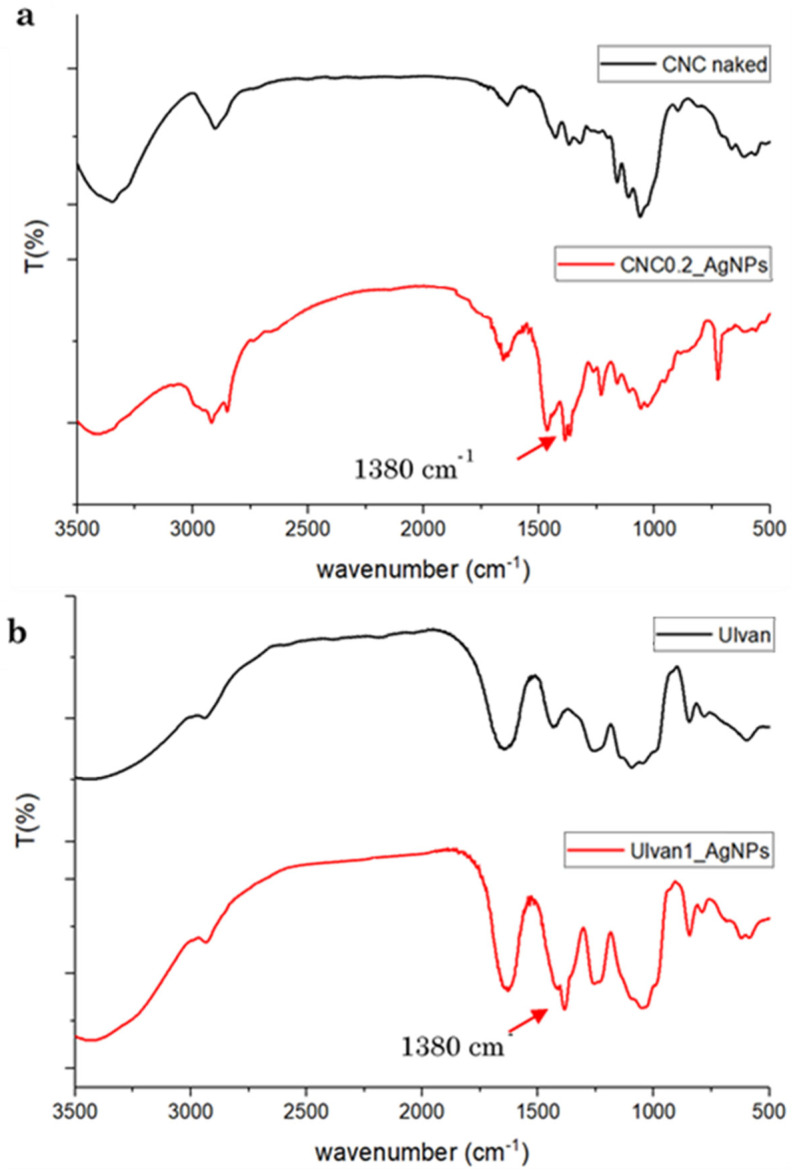
(**a**) FT-IR spectra of CNC0.2_AgNPs and pristine CNC. (**b**) FT-IR spectra of Ulvan1_AgNPs and pristine Ulvan.

FT-IR analysis was employed to identify the functional groups of polysaccharides which should be involved in the stabilization of AgNPs (Figure 5). The comparison between the FT-IR spectra of dried polysaccharides and the developed formulations revealed the presence of a peak at 1380 cm^−1^, attributed to the shift of the peak corresponding to the bending vibration of the carboxyl group of the polysaccharide involved in the stabilization of AgNPs (Figure 5a,b). These results indicated the carboxyl groups of both polysaccharides as responsible for the stabilizing activity [32].

Conclusively, the morphological characterization and the preliminary results of colloidal stability obtained through zeta potential analysis indicated CNC0.2_AgNPs and Ulvan1_AgNPs samples as the most promising and comparable colloidal systems for the development of hybrid hydrogels.

### 2.2. AgNPs Stability in Physiological Environment

**Figure 6:**
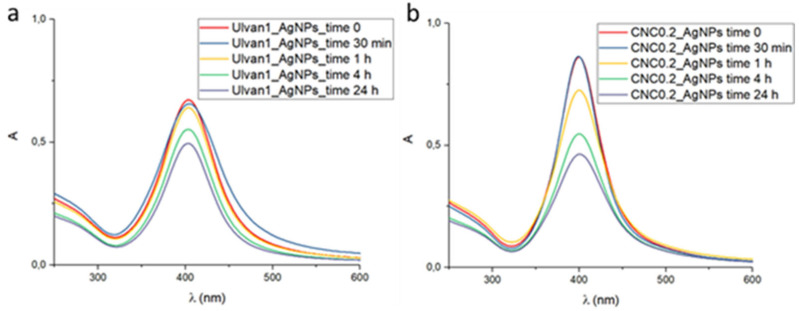
(**a**) UV-vis spectra at different time points of Ulvan1_AgNPs after suspension in PBS 1X. (**b**) UV-vis spectra at different time points of CNC0.2_AgNPs after suspension in PBS 1X. (**a**) Ulvan1_AgNPs; (**b**) CNC0.2_AgNPs.

The tendency of selected AgNP formulations to aggregate under physiological conditions was evaluated by UV-vis analysis at different times after 1:10 dilution of an aqueous colloidal suspension in Phosphate Buffer Saline (PBS) (1× pH 7.4) (Figure 6a,b). The shape of UV-vis spectra of both kinds of synthesized AgNPs was not affected by the changing of ionic strength due to the presence of PBS salts. A high decrease of absorbance experienced by CNC0.2_AgNPs over time was observed. In this case, the precipitated solid was hardly re-suspended due to the intrinsic low stability of CNCs in PBS solution. 

Conversely, the decrease of absorbance recorded for Ulvan1_AgNPs was lower with respect to CNC0.2_AgNPs formulation, and the precipitated aggregates found after 24 h were easily resuspended by shaking the mixture. The recorded decrease of absorbance of AgNPs with time was probably due to polysaccharide aggregation, which can be easily reversed by straightforward re-dispersion of the precipitate. However, the strong stabilizing activity of both polysaccharides did not allow obtaining irreversible aggregates.

### 2.3. AgNPs Antibacterial Activity

**Figure 7:**
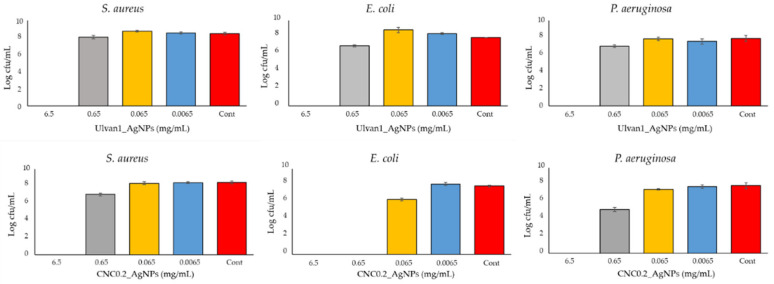
Antibacterial activity of Ulvan1_AgNPs and CNC0.2_AgNPs against *Escherichia coli* ATCC 25,922, *Pseudomonas aeruginosa* ATCC 9027 and *Staphylococcus aureus* ATCC 25,923 after 24 h of incubation in PBS supplemented with 1% TSB and increasing concentration of each AgNPs species (from 0.0065 to 6.5 mg/mL). Data are reported as mean ± standard deviation of at least three independent experiments. Cont: bacteria growth in 1% TSB.

The antibacterial activity of Ulvan1_AgNPs and CNC0.2_AgNPs was evaluated against both Gram-negative (*Escherichia coli* ATCC 25,922 and *Pseudomonas aeruginosa* ATCC 9027) and Gram-positive (*Staphylococcus aureus* ATCC 25,923) bacteria. Time-kill studies were performed to each bacterial strain, using eradicating concentrations of Ulvan1_AgNPs and CNC0.2_AgNPs as reported in Figure 7. Developed nanosystems displayed a comparable activity at tested AgNPs concentrations suggesting a similar mechanism of action. However, *E.coli* strains seems to be more susceptible to CNC0.2_AgNPs presence since a complete eradication was observed even at a lower concentration [0.65 mg/mL].

### 2.4. Incorporation of the Ag Nanocomposites into HA Hydrogels

The presence of carboxylic acid, primary and secondary hydroxyl, and N-acetyl groups into hyaluronic acid (HA) structure has allowed the development of several procedures of functionalization [24]. In the present research, the performed HA modifications followed a well-known protocol for methacrylated HA (Met-HA) preparation [25]. The methacrylation degree, defined as the percentage of methacrylated disaccharidic units, was determined to be 10.2 ± 1.2% by analysis of the Met-HA sample through ^1^H-NMR. According to the previous work reported by Almeida and co-workers, the obtained degree of substitution represents an optimal condition for the development of stable hydrogels [25].

**Figure 8:**
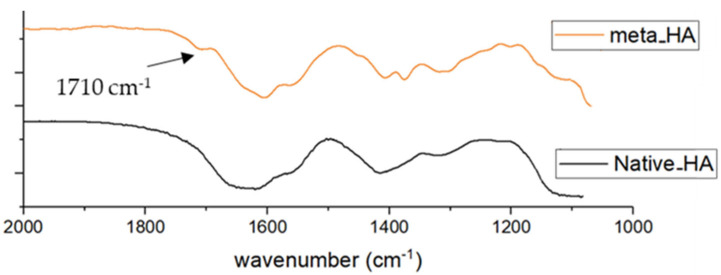
FT-IR spectra of methacrylated HA (Met-HA, orange), and native HA (black).

The FT-IR analysis showed a defined peak at 1710 cm^−1^ in the spectrum of Met-HA corresponding to the C = O stretch of carbonyl ester group, confirming the methacrylation of the polysaccharide (Figure 8, orange line). 

The AgNPs species herein developed were incorporated in photocrosslinkable Met-HA hydrogels. The photo-crosslinking polymerization strategy led to the formation of stable hydrogels through all tested experimental conditions, with a defined 3D shape that was maintained in PBS 1X at 37 °C for 24 h. Hydrogels displayed the initial yellow-orange color of the AgNPs colloidal suspension, and an increase of hydrogel color intensity was observed by increasing the silver concentration (Table 2).

**Figure 9:**
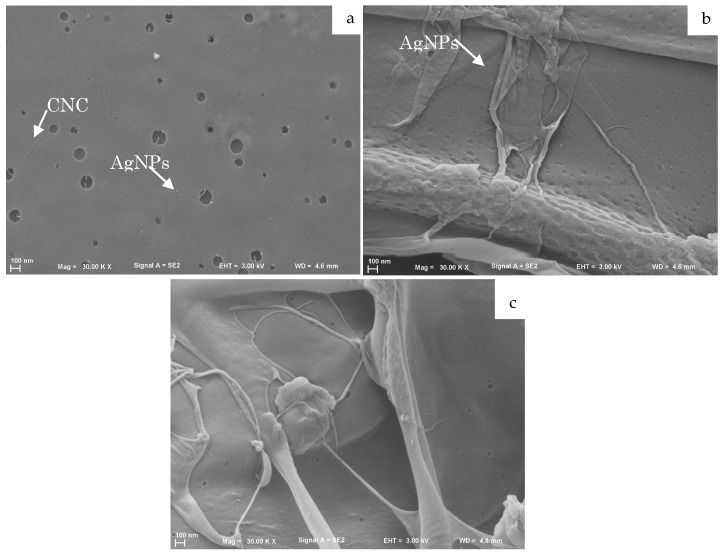
Cross-sectional SEM micrographs of hydrogels incorporating (**a**) CNC0.2_AgNPs and (**b**) Ulvan1_AgNPs. (**a**) Met-HA-CNC0.2_AgNPs; (**b**) Met-HA-Ulvan1_AgNPs; (**c**) Met-HA-Unload.

A preliminary evaluation of the hydrogels’ stability in physiological conditions was performed by immersing them into 1 mL of PBS solution (1×, pH 7.4) for 24 h. No macroscopic changes were observed for all developed hydrogels after their incubation in the simulated physiological environment. All the formulations maintained their initial shape, stiffness, and color. The morphology of hyaluronic acid hydrogels and the possible influence of AgNPs incorporation on their microstructure were investigated by means of SEM analysis. Cross-sections of HA hydrogels were obtained by fracturing lyophilized samples after their immersion in liquid nitrogen. Images relevant to the cross-sections analyzed by SEM are shown in Figure 9. Some brighter spherical shape nanostructures were observed only in loaded hydrogels, suggesting the presence of AgNPs entrapped within the hydrogel’s matrix (Figure 9a,b). In addition, the hyaluronic acid matrix seems not to be negatively affected by AgNPs loading.

## 3. Discussion

In the reported study, two natural sources-derived polymers were investigated as stabilizing agents for the preparation of AgNPs with potential application as antimicrobial agents for wound dressing hydrogels. AgNPs-loaded hydrogels, thanks to the unique nature of silver ions, typically own a strong and fast antimicrobial activity, as well as improved mechanical properties. However, the loading of AgNPs, and any metallic nanocomposites in general, into hydrogel matrices presents several difficulties due to the low stability of metal colloids in physiological/aqueous environments, which could lead to the loss of the nanostructured conformation and thus loss of bioactivities. Indeed, low colloidal stability of metal nanostructures lead to the formation of an inactive and or toxic device whose application into the human body could cause severe side effects to the patients [21].

To this aim, metal colloid and hydrogel stability represent fundamental aspect. Two macromolecular stabilizing agents, i.e., ulvan or cellulose nanocrystals (CNCs), were employed for the fabrication of stable AgNPs suspension with the aim of individuating the most promising formulations in terms of colloidal stability. In this context, we synthetized highly stable silver nanostructures through an easy and reproducible method based on the good stabilizing action of selected polysaccharides, whose supramolecular structures allow for stabilization of metal colloids through the combination of weak interactions and steric hindrance.

In order to investigate the influence of the stabilizer over the biological activity of formed hydrogels, several experimental conditions, such as stabilizing agent and silver nitrate concentrations, were tested to obtain similar colloidal suspensions in terms of silver core size, shape and colloidal surface charge. A preliminary investigation over AgNPs inorganic core size and shape was conducted by comparing the obtained surface plasmon resonance bands as reported in Figure 2. The inorganic core sizes of selected AgNPs formulations (CNC0.2_AgNPs and Ulvan1_AgNPs) were calculated by their XRD pattern using Debye–Scherrer’s equation [30] and were 8.9 and 7.7 nm, respectively. Their inorganic core size and shape were confirmed consequently by morphological investigations by means of STEM and AFM analysis. Stabilizing agents’ activity against particles aggregation has been evaluated in simulated physiological conditions. In our study, ulvan polysaccharide showed a better stabilizing action in comparison to CNCs; this result may be the consequence of different factors such as the intrinsic lower stability of CNCs in PBS and the different interaction between polysaccharides and silver core. Indeed, while the inorganic core in CNC0.2_AgNPs was adsorbed and stabilized over the surface of the crystalline nanorods as indicated by STEM and AFM images, Ulvan1_AgNPs demonstrated to assume a core-shell conformation where the silver core is surrounded by the presence of a thick ulvan coating, as reported by the DLS analysis [32]. However, no particular differences in terms of particles stability were observed after their loading into photocrosslinked methacrylated hyaluronic acid (met-HA).

In order to evaluate their antimicrobial activity, both AgNPs were tested against pathogenic bacteria commonly detected in wound skin infections [44]. CNC0.2_AgNPs and Ulvan1_AgNPs displayed a strong and fast antimicrobial activity against both Gram+ and Gram- bacteria, as reported in Figure 7. Different AgNPs concentrations were tested to evaluate the nanoparticle’s behavior during the formation of the hydrogels. In particular, CNC0.2_AgNPs seems to be more effective against *E. coli* strains, indicating a stronger eradication action compared to Ulvan1_AgNPs that, on the other hand, exhibited higher stability. Such differences in terms of antimicrobial activity may be related to the crystalline structure and rod-like shape of the cellulose nanocomposites with respect to ulvan formulations, since cellulose nanocrystals did not present inherent antimicrobial activity and the net surface charge of both formulations resulted identical [45].

**Figure 10:**
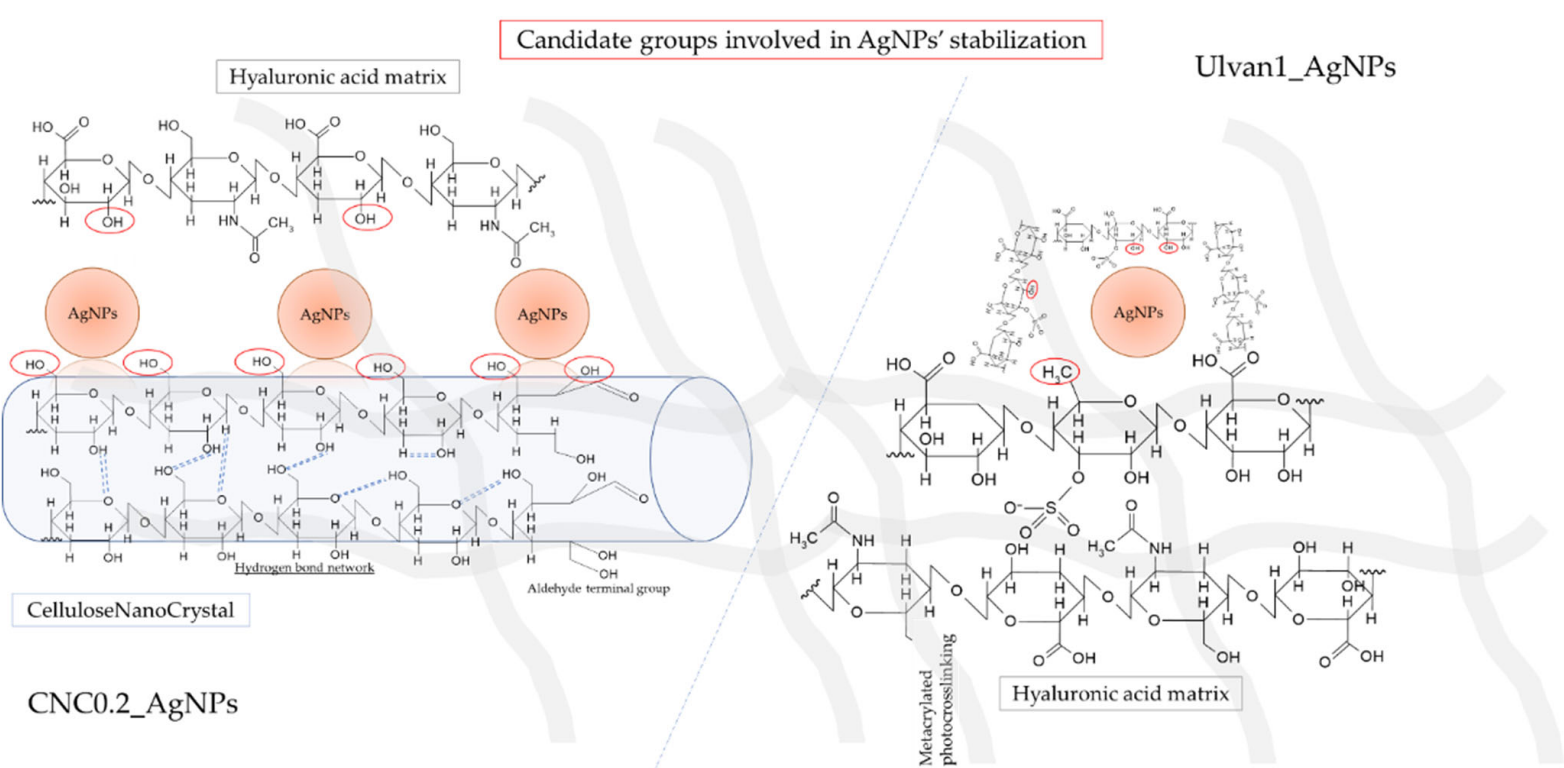
Schematic representation of the developed hydrogels systems. Candidate groups involved in AgNPs interactions have been underlined in red.

Finally, the feasibility of loading developed nanostructures within the polymeric matrix of a potential hydrogel for wound dressing was investigated. Conversely to the lower stability of CNC0.2-AgNPs in simulated physiological media suspension, compared to Ulvan1-AgNPs, no differences in terms of particles stability were observed after loading into the hyaluronic acid polymeric matrix (Figure 9). Morphological investigation performed by SEM analysis highlighted the presence of nanostructures in the polymeric matrix (Figure 9). However, the obtained SEM micrographs may represent only an artifact of hydrogels’ real morphology since before the analysis they underwent different procedures, such as lyophilization, which may strongly affect their microscopical structure. The high stability demonstrated by the developed AgNPs as aqueous suspension is a fundamental aspect to obtain a well-dispersed nanostructured phase in a continuous polymeric matrix. The formed HA-based matrix was an optimal environment in which to maximize nanoparticles stability and dispersion due to the presence of several chemical groups allowing the formation of further weak interactions with AgNPs, such as primary, secondary hydroxyl and N-acetyl groups. A schematic representation of both developed systems is reported in Figure 10.

The strong and fast antibacterial activity displayed by both AgNPs against model pathogenic bacteria was combined with the good stability of the whole system, even when incorporated into polysaccharide matrices, posing the basis for future studies for the preparation of novel antimicrobial devices. In particular, CNC0.2-AgNPs antimicrobial properties against pernicious bacteria such as *E. coli* should be further explored as an alternative to the use of antibiotics in recurrent wound infections.

## 4. Materials and Methods

### 4.1. Materials

Ulvan polysaccharide powder extracted by *Ulva armoricana* was provided by ELICITYL (France) (molecular weight, from 90 to 500 kDa). Microcrystalline cellulose powder (MCC), sodium periodate (NaIO4), hyaluronic acid (Mw = 1.5-1.8 MDa), Pluronic acid F-127, Bovine Serum Albumin, 2-Hydroxy-4′-(2-hydroxyethoxy)-2-methylpropiophenone (Irgacure D-2959), methacrylic anhydride and phosphate buffered saline (PBS) were all purchased from Sigma-Aldrich, Missouri, St. Louis, (USA). Sodium hydroxide and hydrochloric acid were purchased from VWR, Fontenay-sous-Bois (France). Concentrated sulfuric acid (95−98%) was purchased from Laborspirit, Santo Antão do Tojal (Portugal). Sodium hyaluronate (Mw = 253 kDa) purchased from Lifecore Biomedical, Minnesota, Chaska (USA).

### 4.2. Synthesis and Characterization of AgNPs

#### 4.2.1. Synthesis of AgNPs by Sodium Borohydride Reduction in Presence of Polysaccharide Stabilizing Agents

Cellulose nanocrystal (CNCs) were previously isolated from microcrystalline cellulose (MCC) by sulfuric hydrolysis following standard protocols established in our facilities [24].

In a 50 mL flask equipped with magnetic stirring a selected amount of stabilizing agent, i.e., CNCs or ulvan (Table 1), was added to 20 mL of AgNO_3_ water solution (0,17 mg/mL corresponding to 0.107 mg/mL to Ag). After the complete dissolution of the stabilizing agent, 100 μL of an aqueous solution of NaBH_4_ (25 mg/mL) were added to the obtained mixture. The addition of the reducing agent leads to an instantaneous color change from colorless to colored (yellow, orange or brown depending on the type and amount of stabilizing agent). AgNPs formation was detected by UV analysis by monitoring the intensity of the surface plasmon resonance (SPR) bands of AgNPs. The effect of the stabilizing agents used, and their concentration, was assessed by adjusting the stabilizing agent/silver (wt/wt) ratio according to the values reported in Table 3.

The obtained suspensions were dialyzed against deionized water using a dialysis tube (MWCO: 14 KDa) for 2 h, replacing water 3 times. At the end of the reaction, the obtained dispersions were lyophilized for 24 h in order to obtain the dried products for physicochemical characterization.

#### 4.2.2. AgNPs Physical-Chemical Characterization

Obtained colloidal suspensions were characterized by means of UV-Vis spectroscopy recording the wavelength region 200–600 nm at room temperature by using a UV-Vis spectrophotometer (UV-1601, Shimadzu, Japan). Dynamic light scattering (DLS) measurements were performed in 1 cm polystyrene cells at an angle of 173° on a Malvern NanoZS (Malvern, UK) using a He–Ne laser with a wavelength of 633 nm. The CONTIN algorithm (intensity weighted) was used to obtain the average particle size distribution. The average hydrodynamic radius (Rh) and dispersity index (PDI) were calculated by fitting the correlation function with the cumulant method. Zeta potential analysis was carried out using Zetasizer (Nano ZS, Malvern, UK). Colloidal suspensions were transferred into a Folded Capillary cell (DTS1060 Malvern, UK) and analyzed in triplicate at 25 °C, adjusting the pH to 7.4. Fourier Transform Infrared (FTIR) spectroscopy spectra were recorded as KBr pellets (1/80 mg) in the range 4000–400 cm−1 by using IR-Prestige-21 (Shimadzu, Japan). X-ray diffraction patterns (XRD) were obtained using a Bruker D8 ADVANCE X-ray Diffractometer (Bruker, Madison, WI, USA) (wavelength λ = 1.541 Å for Cu Kα radiation) operating at 40 kV and 40 mA. The scanning rate was 0.02° with a scattering angle ranging from 10° to 90°. The crystallinity index of cellulose nanocrystal of CNC0.2_AgNPs formulation was measured using Segal’s method (Equation (1)):

cellulose crystallinity index (%) = ((I_(200) − I_amoprhous))/(I_(200)) × 100(1)

#### 4.2.3. AgNPs Morphological Characterization

AgNPs suspensions were dropped on single-side-polished silicon support for scanning electron microscopy (SEM) analysis, or copper grids coated with a carbon film for transmission electron microscopy (STEM) analysis and dried at room temperature. AgNPs for SEM analysis were coated with a thin film (5 nm) of Au-Pd (80–20 weight %) in a high-resolution sputter coater, 208HR (USA), coupled to an MTM-20 Cressington High-Resolution Thickness Controller. Morphological analysis of dried suspension was conducted by collecting secondary electron (SEM) or transmitted electrons (STEM) images by using an Ultrahigh-resolution Field Emission Gun Scanning Electron Microscope (FEG-SEM), NOVA 200 Nano SEM, FEI Co. (USA). The obtained micrographs were elaborated using ImageJ software and the particles size distribution was measured.

AFM analysis was carried out using L018W46 (Dimension Icon, Bruker, France), depositing single drops of the suspension on a microscope glass slide and allowing water evaporation at room temperature (24 °C). Scans were acquired in SCANASYST-AIR mode, with non-contacting silicon tips on nitride lever from Nanosensor (Switzerland). Images obtained were elaborated using Nanoscope software and the particles size distribution was calculated. At least 150 nanoparticles were selected from different acquired images.

#### 4.2.4. AgNPs Stability in Physiological Condition

Colloidal stability of most promising AgNPs formulations were investigated by UV-Vis analysis of the relevant dispersions in PBS 1X (pH = 7.4) obtained through the dilution of 200 μL of the synthesized AgNPs water dispersions in 1.8 mL of PBS 1X. Nanoparticles stability was monitored over time by recording the shape and absorbance of the surface plasmon resonance (SPR) bands of silver nanoparticles at regular time intervals.

#### 4.2.5. AgNPs Suspension Antibacterial Activity

The minimal inhibitory concentration of ULV-AgNPs and CNC-AgNPs was evaluated against both Gram- (*Escherichia coli* ATCC 25922 and *Pseudomonas aeruginosa* ATCC 9027) and Gram+ (*Staphylococcus aureus* ATCC 25923) bacteria. Bacterial cultures were grown in Tryptone Soya Broth (TSB; Oxoid, UK) medium at 37 °C overnight with agitation (150 rpm). Cells were centrifuged at 9000 rpm for 5 min at 4 °C, and the bacterial pellet was washed twice with sterile PBS (pH 7.4). Bacterial cells were re-suspended in sterile PBS (pH 7.4) with 2% TSB (*v*/*v*) and adjusted to an optical density of 0.05 (λ = 610 nm, with a final density of 3.1 × 10^6^ CFU/mL, 2.6 × 10^6^ CFU/mL, and 1.2 × 10^6^ CFU/mL of E. coli, P. aeruginosa and S. aureus, respectively). A volume of 50 μL of the bacterial suspensions was added to 50 μL of ULV-AgNPs and CNC-AgNPs at different concentrations (0, 0.0065, 0.065, 0.65, and 6.5 mg/mL). Samples were incubated at 37 °C with agitation (150 rpm) for 24 h. Serial dilutions were made, spread onto Tryptone Soya Agar (TSA; Oxoid, UK), and incubated overnight at 37 °C. The minimal inhibitory concentration of ULV-AgNP and CNC-AgNP was determined as the log the colony forming units (CFUmL^−1^). Each experiment was performed in duplicate.

### 4.3. Synthesis and Characterization of Hyaluronic Acids Hydrogels

#### 4.3.1. Synthesis of Methacrylate Modified Hyaluronic Acid (Met-HA)

10-fold molar excess of methacrylic anhydride (MA) was added to a HA water solution (1 wt%, 100 mL). The pH was consequently adjusted between 8 and 8.5 with 5M NaOH added dropwise. Methacrylate-modified hyaluronic acid (Met-HA) was subsequently precipitated using cold ethanol (at −20 °C). The precipitate was collected by centrifugation at 2000× *g*, 5 min. Then the precipitated product was re-dissolved in deionized H_2_O and dialyzed against ultrapure water using a dialysis tube (MWCO: 14 KDa) for 7 days, replaced 3 times per day, in order to remove all unreacted reagents and ethanol. Finally, the solution was filtered (0.45 μm), frozen at −80 °C, and freeze-dried for 24 h in order to obtain a dry product.

#### 4.3.2. Met-HA Chemical Characterization

FT-IR spectroscopy (IR-Prestige-21, Shimadzu, Japan) was used to record the infrared spectra of HA and modified-HA. Spectra were obtained in the range of 400 to 4000 cm^−1^ at a 4 cm^−1^ resolution with 32 scans. ^1^H NMR spectra were recorded with a Varian Inova 500 (USA) at 70 °C. HA solutions were prepared for analysis by dissolving 5 mg of HA derivative in 1 mL of D_2_O.

#### 4.3.3. Development of Hybrid Organic/Inorganic HA-Hydrogels through Photo-Crosslinking Polymerization (Met-HA)

Met-HA-AgNPs hydrogels loaded with selected AgNPs formulations were obtained through photo-crosslinking polymerization of Met-HA. Data of specimen experiments are reported in Table 2. For unloaded-HA hydrogel: 100 μL of Met-HA (1.5% *w*/*v*) and Irgacure D-2959 photoinitiator (1.5% *w*/*v*) water solution was exposed for 50 s under UV light at 365 nm (Table 4, Unloaded-Met-HA). For AgNPs-loaded HA hydrogels, the initial solution composed by Met-HA (1.5% *w*/*v*) and Irgacure D-2959 photoinitiator was loaded with different amounts of AgNPs suspension and exposed to the same conditions employed for the unloaded Met-HA hydrogel.

#### 4.3.4. HA-Hydrogel Microstructure Characterization

The morphology of the photo-crosslinked HA hydrogels was analyzed using SEM. The obtained hydrogels were frozen in liquid nitrogen and freeze-dried for 24 h. Cross-sections of the HA hydrogels were obtained by fracturing lyophilized samples after their immersion in liquid nitrogen. Before analysis, samples were coated with a thin film (5 nm) of Au-Pd (80–20 weight %).

## 5. Conclusions

The potential of CNCs and ulvan, abundant natural-origin polysaccharides, as stabilizing agents for the development of easy and reproducible methodologies of novel AgNPs was investigated.

The developed systems exhibited strong antimicrobial activity combined with good stabilization of silver colloids in PBS. In particular, CNC0.2_AgNPs formulation exhibited stronger antimicrobial activity against Gram-strains.

The unprecedently developed procedure of synthesis of hybrid hydrogels represents a promising alternative method for secure and efficient loading of metal nanostructures into polymeric matrix combining good biocompatibility, provided by the use of polysaccharides from natural sources with proven cytocompatibility, and strong antimicrobial action.

Overall, the developed hybrid hydrogels could represent an innovative formulation for the synthesis of hydrogels with antimicrobial properties for medical treatment of wound healing in order to avoid bacteria invasion, a common but not yet solved health issue.

## Figures and Tables

**Table 1:** Particle size distribution of selected formulations recorded by DLS and relevant δ-potential values.

Sample	Diameter (nm)	Size Distribution Graph	Zeta Potential (mV)
CNC0.2 AgNPs	188.8 ± 18.5 P.I.: 0.89	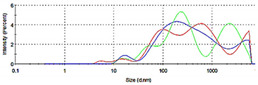	−37.2 ± 2.8
Ulvan1 AgNPs	70.8 ± 11.2 P.I.: 0.35	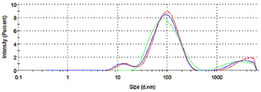	−37.0 ± 0.6

**Table 2:** Macroscopical aspect of the obtained hydrogels at the correspondent Ag weight percentage.

Ag (wt%)	Met-HA-CNC0.2_AgNPs	Met-HA-Ulvan1_AgNPs
Unloaded	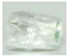	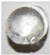
0.00015	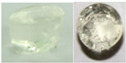	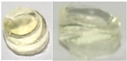
0.0015	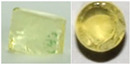	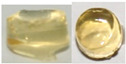
0.00535	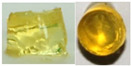	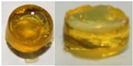
0.0107	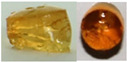	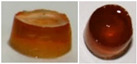

**Table 3:** Peak maxima recorded in the UV spectra of synthesized AgNPs formulations.

Stabilizing Agent	Tested Concentration (mg/mL)	λ Max of Absorption Time 0 (nm)
Cellulose NanoCrystal (CNC)	0.2	unstable
	0.20	399
	0.25	398
	0.50	397
	1	401
Ulvan	0.25	392
	0.50	396
	1	400

**Table 4:** Summary of conditions used for the development of Met-HA hydrogels.

Formulation	Initial Solution	AgNPs Suspension
Unloaded-Met-HA	HA metacrylate (1.5% *w*/*v*) Irgacure D-2959 (0.15% *w*/*v*)	/
Met-HA-CNC_AgNPs	HA metacrylate (1.5% *w*/*v*) Irgacure D-2959 (0.15% *w*/*v*) CNC0.2_AgNPs	(Ag: 0.0107% *w*/*v*) (Ag: 0.00535% *w*/*v*) (Ag: 0.0015% *w*/*v*) (Ag: 0.00015% *w*/*v*)
Met-HA-Ulvan_AgNPs	HA metacrylate (1.5% *w*/*v*) Irgacure D-2959 (0.15% *w*/*v*) Ulvan1_AgNPs	(Ag: 0.0107% *w*/*v*) (Ag: 0.00535% *w*/*v*) (Ag: 0.0015% *w*/*v*) (Ag: 0.00015% *w*/*v*)

## Data Availability

The data presented in this study are available on request from the corresponding author. The data are not publicly available due to privacy issues.
